# Differential Effects of Up- and Down-Regulation of SMR Coherence on EEG Activity and Memory Performance: A Neurofeedback Training Study

**DOI:** 10.3389/fnhum.2020.606684

**Published:** 2020-12-23

**Authors:** Silvia Erika Kober, Christa Neuper, Guilherme Wood

**Affiliations:** ^1^Institute of Psychology, University of Graz, Graz, Austria; ^2^BioTechMed-Graz, Graz, Austria

**Keywords:** cognition, connectivity, mental strategy, neurofeedback, resting EEG

## Abstract

Modulating connectivity measures in EEG-based neurofeedback studies is assumed to be a promising therapeutic and training tool. However, little is known so far about its effects and trainability. In the present study, we investigated the effects of up- and down-regulating SMR (12–15 Hz) coherence by means of neurofeedback training on EEG activity and memory functions. Twenty adults performed 10 neurofeedback training sessions in which half of them tried to increase EEG coherence between Cz and CPz in the SMR frequency range, while the other half tried to down-regulate coherence. Up-regulation of SMR coherence led to between- and within-session changes in EEG coherence. SMR power increased across neurofeedback training sessions but not within training sessions. Cross-over training effects on baseline EEG measures were also observed in this group. Up-regulation of SMR coherence was also associated with improvements in memory functions when comparing pre- and post-test results. Participants were not able to down-regulate SMR coherence. This group did not show any changes in baseline EEG measures or memory functions comparing pre- and post-test. Our results provide insights in the trainability and effects of connectivity-based neurofeedback training and indications for its practical application.

## Introduction

Since the 1960's (Kamiya, 1969), neurofeedback (NF) is used to learn to control one's own electrical brain activity through real-time feedback of EEG signals (Gruzelier, 2014a). Modulating the EEG activity in a desired direction is associated with improvements in behavior, cognitive function, motor function, or mood (Kropotov, 2009; Gruzelier, 2014a).

In the majority of NF training studies, participants receive feedback on changes in the amplitude of a specific EEG oscillation recorded over a specific electrode position (Keizer et al., 2010; Doppelmayr and Weber, 2011; Gruzelier, 2014a; Kober et al., 2015a,b, 2017a; Enriquez-Geppert et al., 2017). For instance, up-regulating the amplitude of the sensorimotor rhythm (SMR, 12-15 Hz) over central brain areas (e.g., electrode position Cz) is associated with an improved stimulus processing capability leading to improvements in cognitive performance in healthy individuals as well as neurologic patients (Gruzelier, 2014a; Kober et al., 2015a,b). However, although specific EEG oscillations recorded over specific brain regions are trained in prior NF studies, concomitant changes in other EEG oscillations and other brain regions are reported as well (Gruzelier, 2014a,b; Kober et al., 2015b, 2017b; Reichert et al., 2016; Ros et al., 2019). This indicates that it is not possible to modulate only one EEG frequency over a specific EEG electrode without modulating also the activity in the networks the trained brain regions are connected with (Ninaus et al., 2013, 2015; Wood et al., 2014; Emmert et al., 2016; Davelaar, 2018; Mayeli et al., 2019).

EEG coherence is a measure of functional connectivity of segregated brain regions. Generally, a functional relationship between two different brain areas is associated with synchronous electrical activity in these two areas. A quantitative measure for this synchrony is the EEG coherence between EEG signals recorded from electrode pairs as a function of frequency (Varela et al., 2001). Many psychiatric and neurological disorders are associated with abnormal brain connectivity (Broyd et al., 2009; Stam, 2014; Babiloni et al., 2016; Shim et al., 2018). Regardless of the task participants are asked to perform, healthy cognitive functioning also activates a whole network of brain areas instead of only a single brain region (Astolfi et al., 2013; Hata et al., 2016; Toppi et al., 2017; Shen et al., 2018). Hence, directly modulating brain connectivity using NF training is potentially useful as therapeutic approach and might improve cognitive function as well (Elmer and Jäncke, 2014; Yamashita et al., 2017). Elmer and Jäncke (2014) maintain that EEG signals recorded from only one specific electrode position are composed of miscellaneous and unspecific brain activity originating from several brain regions. They argue that it might be more efficient to dynamically change functional brain connectivity between specific brain areas of interest instead of modulating one EEG oscillation recorded over one electrode position. Changing brain connectivity by means of NF training takes the dynamic and interconnected nature of the brain into account (Elmer and Jäncke, 2014).

Most connectivity-based NF training studies are real-time fMRI studies (Broyd et al., 2009; Watanabe et al., 2017; Yamashita et al., 2017). EEG-based NF training studies providing feedback about EEG coherence or connectivity values are lacking (Mottaz et al., 2015, 2018; Kajal et al., 2017). According to Gruzelier (2014b): “*As yet little research has been undertaken in examining or training connectivity in EEG neurofeedback, though the approach is being undertaken by practitioners*” (Gruzelier, 2014b, p. 19). For instance, quantitative EEG (QEEG)-based connectivity trainings, in which the individual EEG activity and coherence measures are compared to a normative database and areas that show a hypo- or hyper-coherence are used as target feedback area, are available and in use by practitioners (Walker et al., 2002; Coben et al., 2014, 2018), although there are many open elementary questions, such as:

- Are NF users able to up- and down-regulate EEG coherence voluntarily between two brain regions?
- Does EEG-based coherence training lead to within- or between-session changes in EEG coherence (Klimesch, 1999; Gruzelier, 2014b; Enriquez-Geppert et al., 2017; Ros et al., 2019)?
- Is there a concomitant change in EEG power (in the feedback frequency but also in other EEG frequencies)?
- Does EEG-based coherence training affect cognitive function?

In the present investigation, we addressed these questions. We performed 10 sessions of a connectivity-based NF training in which participants should either increase or decrease the coherence in the SMR frequency range (12–15 Hz) between electrode position Cz and CPz and assessed different cognitive functions in a pre-post-design. We chose EEG positions Cz and CPz because in prior SMR-based NF training studies, in which the SMR amplitude should be increased over Cz, concomitant changes in brain connectivity between Cz and CPz were observed and related to cognitive improvements (Kober et al., 2015b; Reichert et al., 2016). In the present study, we address the question of the equivalence between training the amplitude of a specific EEG frequency or coherence regarding efficiency and direction of outcomes. We hypothesized that directly modulating SMR coherence between these two electrode positions might have effects on SMR amplitude as well as cognitive performance (iii, iv).

Based on prior connectivity NF training studies using real-time fMRI or MEG (Sacchet et al., 2012; Kajal et al., 2017; Yamashita et al., 2017), we expect that participants should be able to modulate the coherence in both directions, that is increasing and decreasing it (i). The question whether the coherence training will lead to changes within the NF training sessions or also to changes between training sessions (ii) is generally an open question in the NF literature (Klimesch, 1999; Gruzelier, 2014b; Enriquez-Geppert et al., 2017; Ros et al., 2019). Klimesch (1999) defines phasic changes in the EEG as event-related changes, which can occur at a rapid rate and which are under volitional control. We would expect to obtain such changes in EEG coherence and/or EEG power within the NF training sessions (within-session changes). Tonic changes in the EEG should be reflected by slower and more stable changes in the EEG, which are less under volitional control than phasic changes (Klimesch, 1999). Tonic changes in the EEG should be reflected by changes in EEG coherence and power values across the NF training sessions (between-session changes) as well as by changes in EEG parameters during a baseline resting condition.

In an additional exploratory approach, we also asked participants to report on their individually used mental strategies during NF training in order to control the feedback bars. These reports were analyzed descriptively to reveal possible relationships between the used mental strategies and the ability to up-/down-regulate SMR coherence. Prior amplitude-based NF studies showed that the used mental strategy might be informative for the NF training success (Nan et al., 2012; Kober et al., 2013, 2017c; Autenrieth et al., 2020).

## Materials and Methods

### Participants

Twenty healthy young adults were pseudo-randomly assigned to two training groups. Five men and five women (mean age = 24.5 years, *SD* = 2.22) performed a SMR coherence up-regulation training while the other half of the participants (5 men and 5 women; mean age = 25.3 years, *SD* = 2.16) performed a SMR coherence down-regulation training. To estimate the expected effect sizes, we calculated the observed effect sizes of one of our previous NF training studies, in which the effects of SMR-based amplitude NF training on cognitive functions in healthy individuals was investigated, and which has a comparable pre-post design as the proposed study (Kober et al., 2015b). In this previous study, we examined two groups with an *N* per group of 10. For this previous study, the observed significant effects were large effects of *f* > 0.40. Power calculations revealed that for the present study, we will reach moderate to high power levels of >80%. Participants were not aware of the grouping design before all measurements were finished. All volunteers gave written informed consent. The study was approved by the local ethics committee of the University of Graz, Austria and is in accordance with The Code of Ethics of the World Medical Association (Declaration of Helsinki) for experiments involving humans (WMA World Medical Association, 2009). Volunteers were paid for their participation (8€ per hour).

### Procedure and Pre-post Assessment of Cognitive Function

For this interventional study, we used a pre-post design. Before the first (pre-test) and after the last (post-test) NF training session, participants performed a cognitive test battery assessing different memory functions since prior SMR-based NF training studies show that increasing the SMR amplitude and concomitantly changing SMR coherence are associated with improvements in memory functions (Gruzelier, 2014a; Kober et al., 2015a,b; Reichert et al., 2016). The Visual and Verbal Memory Test [Visueller und verbaler Merkfähigkeitstest 2 – VVM 2 by Schelling and Schächtele (2001)] was used to assess short- [immediate recall of learned material, time point (1)] and long-term [remember visuo-spatial and verbal material for up to 24 h, time point (2)] memory of visuo-spatial and verbal material. In the subtest “city map” (visuo-spatial memory) participants have to memorize a route drawn on a map and then mark it on the same map during recall. In the subtest “construction” (verbal memory) a description of a building is presented and participants have to learn names, numbers, and propositional contents. Participants have to memorize the learned material for 2 min before the immediate recall phase.

The Digit Span forward test was used to assess number storage capacity (short term memory). Therefore, a series of digits (e.g., '8, 3, 4') is presented followed by immediate recall of these digits. If the recall is correct, participants are provided with an extended list (e.g., '9, 2, 4, 0'). The length of the longest list a person can remember is defined as a person's digit span. In the forward task, participants are required to recall the digits in the given order. The backwards tasks of the Digit Span test, in which participants have to recall the sequence of digits backwards, was used to assess working memory (Schellig, 1993, 2011; Schuhfried, 2011).

The Corsi Block Tapping forward test (CBTT) was used to assess visual short-term memory capacity and implicit visuo-spatial learning. In this test, the participant views nine irregularly positioned blocks on a board and the experimenter taps on a number of these blocks in turn. Afterwards, the participant is required to tap on the same blocks in the same order. In the backwards task of the Corsi Block Tapping test, which assesses working memory, the participant should tap on the blocks in a reverse order. The number of blocks increases by one after three items. When the participant makes an error in three successive items the test stops (Schellig, 1993, 2011; Schuhfried, 2011).

To avoid learning effects available parallel forms of the standardized psychometric tests were used in the pre- and post-measurement. The cognitive test battery was performed on two consecutive days during the pre- and post-test, respectively.

The 10 NF training sessions were performed within 3 to 4 weeks. Before and after the 10 NF training sessions, resting EEG measurements with open eyes with a duration of 1 min each were performed. After the first, fifth, and tenth NF training session, we asked the participants to verbally describe their mental strategies, which they have used to control the feedback bars (Kober et al., 2013, 2017c; Davelaar et al., 2018; Autenrieth et al., 2020).

### Neurofeedback Training

EEG data was recorded by Ag/AgCl passive electrodes (ExG sensors, Mind Media BV) over Cz and CPz using a 10-channel EEG amplifier with a sampling rate of 256 Hz (NeXus-10 MKII, Mind Media BV). A conductive, liquid gel was used to ensure good contact between EEG electrode and skin as well as low electrode impedance. Cz was referenced to the left mastoid and CPz to the right mastoid position (Nolte et al., 2004). The ground was placed on the left mastoid and one EOG channel was recorded over the left eye. The NeXus-10 system has a built-in electrode check. The system uses DC offset checking, which is done online and does not interfere with the signals.

The NF training paradigm was generated with the BioTrace+ software (Mind Media BV). The EEG signal recorded over Cz and CPz was used to calculate the coherence, which is defined as the ratio of the auto-spectra of the two EEG channels and their cross spectra in the EEG frequency domain. Online EEG data processing included an online Fast Fourier Transformation (FFT) of EEG epochs with a length of 1 s. Coherence in the 12–15 Hz frequency range was used as feedback signal. The aim of the coherence up-regulation group was to increase the coherence in the 12–15 Hz range, which is generally associated with synchronous electrical activity over Cz and CPz (Nunez et al., 1997; Varela et al., 2001). The coherence down-regulation group should decrease the coherence in the SMR frequency range, which is associated with desynchronized activity over Cz and CPz.

Each NF session consisted of one baseline run and six feedback runs. Each run took 3 min. Participants received visual feedback. They saw three vertically moving bars on a feedback screen. The height of the bar in the middle of the screen reflected the SMR coherence values between Cz and CPz. The height of the bar on the left and right side of the screen depicted the amplitude of theta power (4–7 Hz) and beta power (21–35 Hz) recorded over Cz, respectively. These two outer bars were used as control bars to prevent the NF users from producing too many artifacts such as blinking, which would lead to slow wave artifacts in the EEG in, e.g., theta frequency range, or producing muscle artifacts, which would have increased high frequency noise in the beta frequency range and, consequently, also SMR activity (Doppelmayr and Weber, 2011; Kober et al., 2015b).

During the initial baseline run, participants watched the moving bars depicting their brain activity in real-time but were instructed to relax and watch but not to control the bars. Based on the data recorded during the baseline run, individual thresholds for the feedback runs were calculated. Mean theta and beta power +1SD was used to set the thresholds for the two outer control bars, mean coherence in the SMR frequency range was used as threshold for the feedback bar in the middle of the screen. The threshold for the bar in the middle of the screen was adapted after each feedback run, while the thresholds for the theta and beta control bars were kept constant over all feedback runs. The thresholds were shown as white horizontal lines, which overlaid the feedback bars.

During the feedback runs, both groups were instructed to keep the outer two bars below their respective thresholds. The coherence up-regulation group was instructed to increase the bar in the middle of the screen above its threshold by using for instance motor imagery. Prior brain-computer interface (BCI) studies showed that motor imagery can lead to increased brain connectivity (Buch et al., 2012). The down-regulation group was instructed to keep the bar in the middle of the screen below its threshold. This should be reached by being mentally focused and physically relaxed. Prior NF studies, in which participants should increase SMR amplitude, which led to a concomitant decrease in brain connectivity, used the same instruction (Kober et al., 2015b; Reichert et al., 2016). Note that the individual reports of participants about their used mental strategies during NF training revealed that participants used many different strategies beside the strategies that were suggested in the instruction (**Figure 4**). If all three bars were at their desired states, the bars' colors turned green and a reward counter shown on the feedback display increased.

### EEG Data Analysis

Offline data preprocessing and analysis were performed with the Brain Vision Analyzer software (version 2.01, Brain Products GmbH, Munich, Germany). Ocular artifacts such as eye blinks were manually rejected by visual inspection based on the information about EOG activity provided by the EOG channel. After ocular artifact correction, automated rejection of other EEG artifacts (e.g., muscles) was performed (Criteria for rejection: >50.00 μV voltage step per sampling point, absolute voltage value > ± 120.00 μV, lowest allowed activity in 100 ms intervals: 0.5 μV). All epochs with artifacts were excluded from the EEG analysis (about 9% of the data).

To analyze EEG coherence in the SMR frequency range, each NF run was cut in artifact free 1-s epochs. FFT transformation was performed per epoch (Hanning window, 10%). Then, the imaginary part of coherence was calculated for the channel pair Cz-CPz and average coherence values in the frequency range of 12–15 Hz were extracted per run. Imaginary coherence assumes that signals with a time-lag are from distinct sources (true connectivity) while signals without a time-lag are due to volume conduction. Hence, we calculated the imaginary part of coherence to avoid that values are affected by volume conductor issues, which is particularly relevant for measurements done with adjacent channels (e.g., Cz and CPz) (Nolte et al., 2004). In addition to the imaginary part of coherence, we also analyzed the magnitude squared coherence, since participants received feedback on this coherence measure during real-time feedback. Results of magnitude squared coherence measure can be found in the Supplementary Material.

To analyze EEG power during the NF training, we extracted absolute power values in the SMR (12–15 Hz), theta (4–7 Hz), and beta (21–35 Hz) frequency range by means of complex demodulation implemented in the Brain Vision Analyzer software (version 2.01) (Draganova and Popivanov, 1999; Brain Products GmbH, 2009). Power values of the artefact free 1-s epochs were averaged per run.

The EEG data of the eyes-open resting measurements were preprocessed and analyzed in the same way as the NF EEG data.

### Statistical Analysis

To evaluate the effects of within- and between-session changes in EEG power and coherence simultaneously in a single statistical model, we employed mixed-effects models with the linear fixed effects session (NF training session 1–10) and run (baseline run and 6 feedback runs) for the dependent variables SMR coherence and EEG power over Cz and CPz (either SMR, theta, or beta power) separately for the SMR coherence up- and down-regulation group (Type I Analysis of Variance with Satterthwaite's method). Subjects, individual regression slopes across sessions and runs were included in the model as crossed random effects (Baayen et al., 2008). Mixed effect modeling was performed in R (Bates et al., 2015), freely available at http://cran.r-project.org. The lme4 package was used (Bates et al., 2015).

SMR coherence assessed during eyes open resting measurements was compared between the pre- and post-test (before and after completing 10 NF training sessions) using paired samples *t*-tests, separately for the SMR coherence up- and down-regulation group. To analyze the cognitive data, paired samples *t*-tests were calculated to compare the pre- and post-test results separately per group. Alpha level was set to *p* = 0.05. Bonferroni correction was applied for multiple testing considering the number of repeated measurements for each cognitive construct. We refrained from calculating correlations between cognitive changes and NF training success since correlations with *N*=10 are not meaningful (Bonett and Wright, 2000).

The individually reported mental strategies to control the feedback bars during NF training were assigned per session (first, fifth, and tenth NF session) to one or more of 11 different categories of responses defined according to Kober et al. (2013) and Autenrieth et al. (2020): “No strategy,” “Relaxing,” “Concentration,” “Visual” (all sorts of visual imagery, mental analogies to the bars, gaze fixation), “Auditory” (all sorts of auditory imagery), “Breath” (conscious breathing, active control of breathing), “Cognition” (subsumes occurrence of thoughts/mental activity, and memories not related to the task), “Cheering” (cheering on bars/oneself), “Body” (focus on the body activity or the activity of any of its parts, facial expressions, levels of tension), “Motor imagery” (imagery of body movements, doing sports), “Emotions” (emotional imagery, thinking of beautiful things). Absolute frequencies of the reported mental strategies were statistically compared between groups using χ^2^ tests. Additionally, average regression slopes were calculated separately for each mental strategy reported during the first, fifth, and tenth NF training session (predictor variable = run number – to reveal within session changes in SMR coherence; dependent variable = SMR coherence between Cz-CPz) per group.

## Results

### NF Performance

#### SMR Coherence

In Table 1, the results of the mixed effect models for the dependent variable SMR coherence are summarized for both groups.

**Table 1:** Results of the mixed-effects model with the linear fixed effects session (NF training session 1–10) and run (baseline run and 6 feedback runs) and the crossed random effects subjects, individual regression slopes across sessions, and runs for the dependent variable SMR coherence during neurofeedback training, presented separately for the coherence up-regulation and down-regulation group.

		**Up-regulation group**			**Down-regulation group**		
		***F (df, dfError)***	***MSE***	***p-value***	***F (df, dfError)***	***MSE***	***p-value***
SMR coherence	Session	6.904 (1, 87.12)	0.017	0.010^*^	0.597 (1.89)	0.001	0.442
	Run	8.443 (1, 58.60)	0.021	0.005^**^	0.003 (1.59)	<0.001	0.955
	Session^*^run	0.109 (1,532.63)	<0.001	0.741	0.494 (1.539)	0.001	0.483

Significant results are marked with * (*p < 0.05; **p < 0.01).

**Figure 1:**
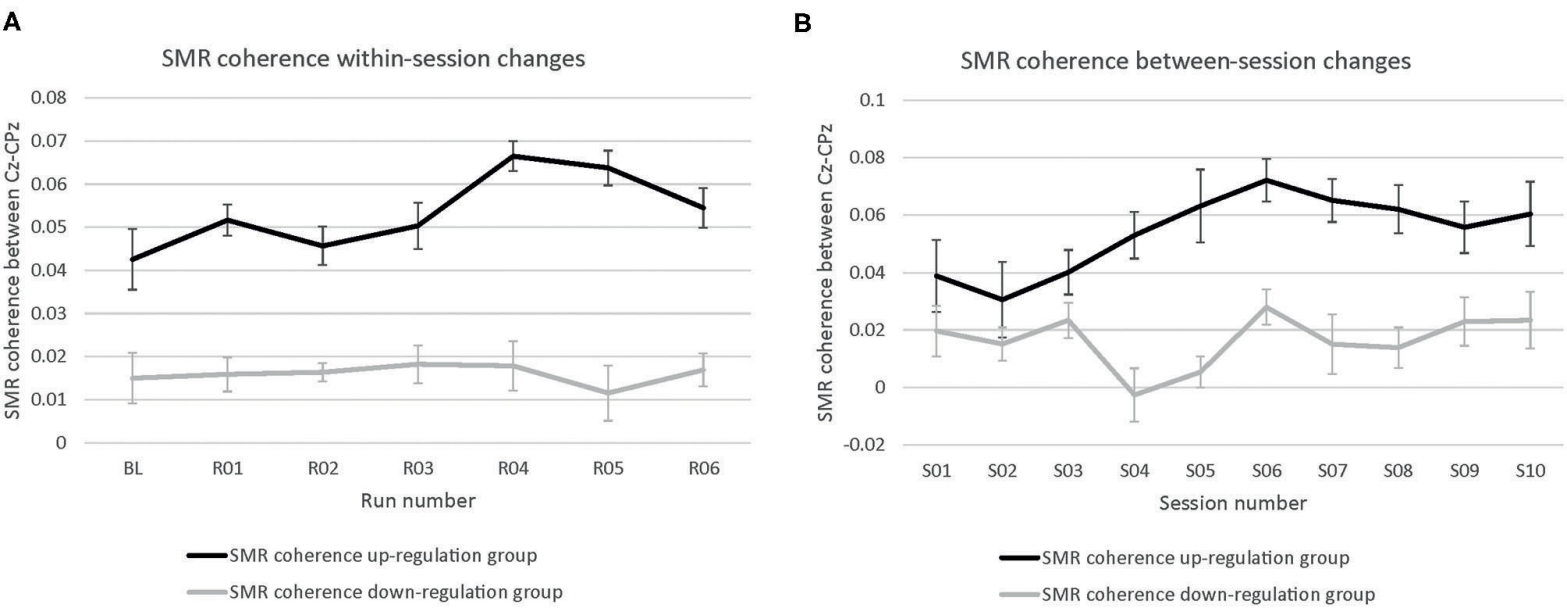
Neurofeedback training performance. Changes in SMR coherence between Cz and CPz **(A)** within neurofeedback training sessions (averaged across all 10 training sessions) and **(B)** between neurofeedback training sessions (averaged across baseline run and six feedback runs per session), presented separately for the coherence up- and down-regulation group. Error bars represent the Cousineau-Morey transformed standard errors.

**Figure 2:**
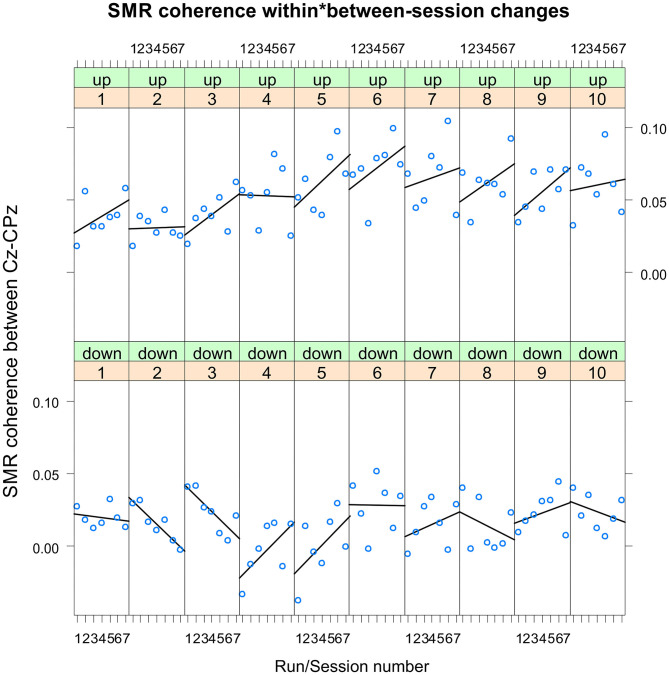
Neurofeedback training performance per training session. Changes in SMR coherence between Cz and CPz across feedback runs (within-session changes, run 1 = baseline run, run 2 = 1st feedback run, run 7 = 6th feedback run), presented separately per neurofeedback training session (1–10) and group (up-regulation group: upper panel, down-regulation group: lower panel).

The coherence up-regulation group showed a linear increase in SMR coherence between sessions (Figure 1B) as indicated by a significant main effect session (Table 1). This might be a sign of between-session changes in EEG coherence in this group. SMR coherence also increased significantly within NF training sessions in the up-regulation group (Figure 1A, significant main effect run). The coherence down-regulation group showed no significant changes in SMR coherence, neither within nor between NF training sessions (Figure 1). Figure 2 shows changes in SMR coherence across feedback runs (within-session changes) separately for each of the 10 NF training sessions.

*T*-tests revealed that groups did not differ in SMR coherence values between the first three NF training sessions (all *p* > 0.32, Figure 1B).

#### SMR Power

**Figure 3:**
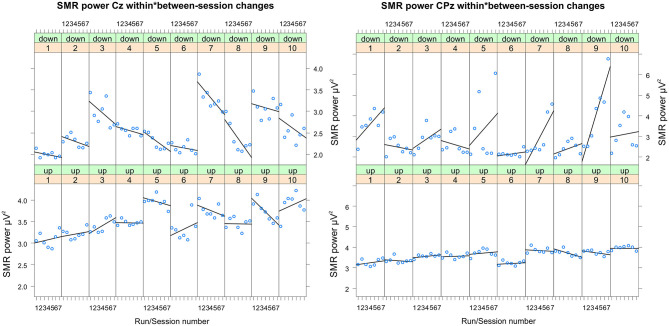
Changes in SMR power recorded over Cz and CPz per training session. Changes in SMR power over Cz (left panel) and CPz (right panel) across feedback runs (within-session changes, run 1 = baseline run, run 2 = 1st feedback run, run 7 = 6th feedback run), presented separately per neurofeedback training session (1–10) and group (down-regulation group: upper panel, up-regulation group: lower panel).

In Table 2, the results of the mixed effect models for the dependent variable SMR power are summarized for both groups and for electrode position Cz and CPz. Table 3 summarizes mean changes in SMR power across NF runs (within-session changes) and Table 4 summarizes mean changes in SMR power across NF sessions (between-session changes). The coherence up-regulation group showed a significant linear increase in SMR power between sessions at both electrode positions, Cz and CPz (Tables 2, 4). In contrast, the coherence down-regulation group showed significant changes in SMR power within NF training session across feedback runs (Tables 2, 3). SMR power increased over CPz and decreased over Cz across feedback runs within NF training sessions (Table 3). Figure 3 shows changes in SMR power across feedback runs (within-session changes) separately for each of the 10 NF training sessions over Cz and CPz.

**Table 2:** Results of the mixed-effects model with the linear fixed effects session (NF training session 1–10) and run (baseline run and 6 feedback runs) and the crossed random effects subjects, individual regression slopes across sessions and runs for the dependent variables SMR power recorded over Cz and CPz during neurofeedback training, presented separately for the coherence up-regulation and down-regulation group.

		**Up-regulation group**			**Down-regulation group**		
		***F (df, dfError)***	***MSE***	***p-value***	***F (df, dfError)***	***MSE***	***p-value***
SMR power Cz	Session	9.476 (1, 87.02)	3.139	0.003^**^	2.627 (1, 88.00)	1.433	0.109
	Run	0.021 (1, 54.25)	0.007	0.885	15.715 (1, 59.60)	8.573	<0.001^***^
	Session^*^run	2.206 (1,528.00)	0.731	0.138	1.053 (1,533.72)	0.575	0.305
SMR power CPz	Session	7.399 (1, 87.01)	1.507	0.008^**^	0.164 (1, 88.01)	1.083	0.686
	Run	0.032 (1, 59.68)	0.007	0.858	6.035 (1, 59.50)	39.786	0.017^*^
	Session^*^run	2.908 (1,532.30)	0.592	0.089	2.145 (1,533.55)	14.141	0.144

Significant results are marked with * (*p < 0.05; **p < 0.01; ***p < 0.001).

**Table 3:** Changes in mean SMR, theta, and beta power recorded over Cz and CPz within neurofeedback training sessions (averaged across all 10 training sessions), presented separately for the coherence up- and down-regulation group and Cousineau-Morey transformed standard errors.

	**Up-regulation group**						**Down-regulation group**					
	**Cz - mean power μV**^****2****^ ***(SE)***			**CPz - mean power μV**^****2****^ ***(SE)***			**Cz - mean power μV**^****2****^ ***(SE)***			**CPz - mean power μV**^****2****^ ***(SE)***		
	**SMR**	**Theta**	**Beta**	**SMR**	**Theta**	**Beta**	**SMR**	**Theta**	**Beta**	**SMR**	**Theta**	**Beta**
Baseline	3.49 (0.08)	9.70 (0.34)	4.32 (0.28)	3.47 (0.08)	8.36 (0.43)	3.68 (0.14)	2.87 (0.12)	10.85 (0.37)	2.81 (0.39)	2.19 (0.33)	8.44 (0.32)	1.87 (0.35)
Run 1	3.61 (0.06)	11.06 (0.13)	4.42 (0.25)	3.66 (0.06)	9.08 (0.11)	3.40 (0.10)	2.58 (0.05)	11.60 (0.39)	2.70 (0.39)	2.63 (0.26)	9.19 (0.28)	2.28 (0.30)
Run 2	3.49 (0.03)	10.77 (0.14)	4.66 (0.16)	3.64 (0.07)	8.84 (0.09)	3.60 (0.11)	2.52 (0.07)	10.75 (0.17)	2.93 (0.39)	3.09 (0.10)	9.14 (0.19)	2.78 (0.39)
Run 3	3.47 (0.03)	10.60 (0.16)	4.69 (0.08)	3.53 (0.02)	8.69 (0.05)	3.64 (0.13)	2.52 (0.03)	10.73 (0.13)	3.21 (0.42)	3.16 (0.18)	8.94 (0.13)	3.01 (0.43)
Run 4	3.45 (0.03)	10.37 (0.14)	4.68 (0.07)	3.54 (0.03)	8.58 (0.15)	3.55 (0.06)	2.48 (0.05)	10.70 (0.22)	2.90 (0.41)	3.04 (0.11)	9.20 (0.37)	2.50 (0.31)
Run 5	3.61 (0.11)	10.32 (0.11)	4.99 (0.22)	3.55 (0.03)	8.39 (0.13)	3.70 (0.09)	2.46 (0.08)	10.66 (0.15)	3.03 (0.44)	2.93 (0.06)	8.30 (0.22)	2.88 (0.38)
Run 6	3.52 (0.05)	10.01 (0.21)	5.09 (0.26)	3.56 (0.04)	8.31 (0.22)	3.78 (0.17)	2.44 (0.05)	10.02 (0.29)	3.27 (0.46)	3.57 (0.34)	8.06 (0.22)	3.18 (0.42)

**Table 4:** Changes in mean SMR, theta, and beta power recorded over Cz and CPz between neurofeedback training sessions (averaged across baseline run and six feedback runs per session), presented separately for the coherence up- and down-regulation group and Cousineau-Morey transformed standard errors.

	**Up-regulation group**						**Down-regulation group**					
	**Cz - mean power μV**^****2****^ ***(SE)***			**CPz - mean power μV**^****2****^ ***(SE)***			**Cz - mean power μV**^****2****^ ***(SE)***			**CPz - mean power μV**^****2****^ ***(SE)***		
	**SMR**	**Theta**	**Beta**	**SMR**	**Theta**	**Beta**	**SMR**	**Theta**	**Beta**	**SMR**	**Theta**	**Beta**
S 01	3.07 (0.20)	9.38 (0.61)	4.57 (0.32)	3.25 (0.25)	8.01 (0.69)	3.91 (0.25)	2.00 (0.23)	9.23 (0.71)	2.18 (0.41)	3.60 (1.01)	7.83 (0.84)	3.34 (0.80)
S 02	3.21 (0.19)	10.36 (0.33)	4.75 (0.39)	3.35 (0.16)	9.28 (1.08)	3.78 (0.28)	2.30 (0.30)	9.28 (0.49)	3.27 (0.58)	2.47 (0.29)	7.89 (0.67)	2.87 (0.87)
S 03	3.41 (0.13)	10.05 (0.51)	4.35 (0.12)	3.56 (0.13)	8.18 (0.51)	3.38 (0.16)	2.97 (0.48)	12.33 (0.99)	2.73 (0.47)	2.87 (0.22)	8.62 (0.70)	2.80 (0.53)
S 04	3.47 (0.09)	10.22 (0.31)	4.28 (0.22)	3.57 (0.10)	9.13 (1.04)	3.40 (0.11)	2.56 (0.37)	11.91 (1.24)	2.81 (0.51)	2.60 (0.34)	8.67 (0.36)	2.42 (0.31)
S 05	3.96 (0.24)	10.65 (1.18)	5.40 (0.59)	3.72 (0.18)	9.07 (0.61)	3.79 (0.23)	2.30 (0.31)	9.13 (1.30)	3.11 (0.72)	3.35 (0.63)	11.35 (2.76)	2.13 (0.43)
S 06	3.33 (0.15)	10.60 (0.30)	5.03 (0.37)	3.22 (0.16)	8.89 (0.82)	3.78 (0.32)	2.15 (0.22)	9.59 (0.71)	2.86 (0.48)	2.15 (0.65)	10.04 (2.20)	2.77 (0.43)
S 07	3.75 (0.16)	11.43 (0.92)	4.55 (0.28)	3.84 (0.11)	8.60 (0.36)	3.78 (0.19)	3.30 (0.43)	11.55 (0.92)	3.29 (0.49)	2.95 (0.22)	8.37 (0.55)	2.99 (0.55)
S 08	3.45 (0.33)	9.84 (1.07)	3.93 (0.48)	3.71 (0.36)	8.43 (1.01)	3.38 (0.37)	2.37 (0.15)	10.74 (0.61)	2.71 (0.41)	2.40 (0.18)	7.38 (0.59)	2.43 (0.35)
S 09	3.73 (0.15)	11.03 (0.50)	4.74 (0.23)	3.74 (0.13)	8.32 (0.46)	3.68 (0.23)	3.08 (0.40)	12.47 (1.48)	3.42 (0.54)	4.09 (1.04)	10.33 (1.66)	2.23 (0.32)
S 10	3.89 (0.44)	9.97 (1.07)	5.62 (0.78)	3.96 (0.39)	8.35 (0.91)	3.54 (0.37)	2.61 (0.22)	11.36 (0.70)	3.54 (0.61)	3.10 (0.40)	8.55 (0.60)	2.31 (0.39)

#### Theta Power

In Table 5, the results of the mixed effect models for the dependent variable theta power are summarized for both groups and for electrode position Cz and CPz.

**Table 5:** Results of the mixed-effects model with the linear fixed effects session (NF training session 1–10) and run (baseline run and 6 feedback runs) and the crossed random effects subjects, individual regression slopes across sessions and runs for the dependent variables theta power recorded over Cz and CPz during neurofeedback training, presented separately for the coherence up-regulation and down-regulation group.

		**Up-regulation group**			**Down-regulation group**		
		***F (df, dfError)***	***MSE***	***p-value***	***F (df, dfError)***	***MSE***	***p-value***
Theta power Cz	Session	2.627 (1, 85.86)	3.934	0.109	3.338 (1, 87.01)	18.115	0.071
	Run	0.563 (1, 57.68)	0.844	0.456	8.178 (1, 59.47)	44.382	0.006^**^
	Session^*^run	5.135 (1,520.15)	7.690	0.024^*^	4.863 (1,527.59)	26.395	0.028^*^
Theta power CPz	Session	0.172 (1, 87.07)	0.309	0.680	0.420 (1, 87.41)	0.936	0.519
	Run	2.176 (1, 59.34)	3.908	0.145	2.556 (1, 59.44)	5.691	0.115
	Session^*^run	3.743 (1,530.15)	6.723	0.053	0.236 (1,530.04)	0.525	0.628

Significant results are marked with * (*p < 0.05; **p < 0.01; ***p < 0.001).

The coherence up-regulation group showed no constant significant changes in theta power neither within nor between NF training sessions (Tables 3, 4). The significant interaction effect session^*^run for electrode position Cz indicates a linear increase in theta power during session 1, while theta slightly decreased or showed no changes during the rest of the NF training sessions (Table 5). No significant effects were observed for CPz (Table 5).

The coherence down-regulation group showed a decrease in theta power within training sessions across feedback runs over Cz (Tables 3, 5). Additionally, the significant interaction effect session^*^run indicates that theta decreased during session 1, 7, 8, and 9, while there were no significant changes in theta power across feedback runs during the other sessions. No significant effects were observed for CPz (Table 5).

#### Beta Power

In Table 6, the results of the mixed effect models for the dependent variable beta power are summarized for both groups and for electrode position Cz and CPz.

**Table 6:** Results of the mixed-effects model with the linear fixed effects session (NF training session 1–10) and run (baseline run and 6 feedback runs) and the crossed random effects subjects, individual regression slopes across sessions and runs for the dependent variables beta power recorded over Cz and CPz during neurofeedback training, presented separately for the coherence up-regulation and down-regulation group.

		**Up-regulation group**			**Down-regulation group**		
		***F (df, dfError)***	***MSE***	***p-value***	***F (df, dfError)***	***MSE***	***p-value***
Beta power Cz	Session	0.534 (1, 87.02)	1.076	0.467	4.267 (1, 79.01)	10.825	0.042^*^
	Run	9.601 (1, 59.23)	19.357	0.003^**^	4.611 (1, 53.76)	11.698	0.036^*^
	Session^*^run	0.901 (1,530.66)	1.816	0.343	0.902 (1,479.87)	2.289	0.343
Beta power CPz	Session	0.933 (1, 87.01)	0.801	0.337	2.347 (1, 79.01)	7.545	0.130
	Run	1.918 (1, 57.68)	1.646	0.172	14.617 (1, 51.98)	46.982	<0.001^***^
	Session^*^run	0.503 (1,529.85)	0.432	0.479	2.126 (1,478.04)	6.834	0.145

Significant results are marked with * (*p < 0.05; **p < 0.01; ***p < 0.001).

The coherence up-regulation group showed a linear increase within NF training sessions across feedback runs in beta power over Cz but not over CPz (Tables 3, 6).

The coherence down-regulation group showed a linear increase in beta power within training sessions across feedback runs over Cz and CPz (Tables 3, 6) as well as an increase in beta power between NF training sessions over Cz (Tables 4, 6).

### SMR Coherence During Resting Measurements

For the up-regulation group, paired samples *t*-tests revealed a higher SMR coherence during the resting measurements after (*M* = 0.02006, *SE* = 0.00542) the 10 NF training sessions compared to the pre-test (*M* = −0.00016, *SE* = 0.00542), which was significant by trend [*t*(9) = −1.87, *p* = 0.09]. The down-regulation group showed no significant changes in resting coherence [pre-test: *M* = 0.00303, *SE* = 0.00620; post-test: *M* = 0.01667, *SE* = 0.00620; *t*(9) = −1.10, *p* = 0.30].

### Cognitive Function

When comparing the results of the cognitive test battery between the pre- (before the 1st NF training session) and post-assessment (after the 10th NF training session), significant improvements were only observed in the coherence up-regulation group (Table 7). The up-regulation group showed significant improvements in short term memory (Digit Span forward, VVM2 construction 1) and working memory (Digit Span backwards) performance after the last NF training session (post) compared to the pre-assessment.

**Table 7:** Means and standard errors of the behavioural data and the results of the statistical analyses (*t*-tests) of the pre-post comparison, separately for each group.

	**Up-regulation group (*****N*** **= 10)**			**Down-regulation group (*****N*** **= 10)**		
	**pre**	**post**		**pre**	**post**	
	***Mean (SE)***		***p-value***	***Mean (SE)***		***p-value***
CBTT forward (raw scores)	10.60 (0.60)	10.30 (0.76)	0.58	10.10 (0.64)	11.20 (0.55)	0.07
CBTT backwards (raw scores)	9.70 (0.52)	10.30 (0.30)	0.17	8.80 (0.53)	9.60 (0.64)	0.12
Digit span forward (raw scores)	7.40 (0.54)	8.90 (0.50)	0.01^*^	8.00 (0.71)	8.60 (0.62)	0.36
Digit Span backwards (raw scores)	7.30 (0.50)	8.30 (0.54)	0.01^*^	8.80 (0.59)	9.00 (0.61)	0.69
VVM2 city map 1 (T-scores)	47.90 (3.26)	53.60 (2.90)	0.14	46.00 (2.81)	52.20 (2.95)	0.04
VVM2 city map 2 (T-scores)	43.80 (3.20)	46.30 (3.23)	0.24	45.50 (3.46)	51.40 (2.70)	0.12
VVM2 construction 1 (T-scores)	42.40 (2.93)	50.20 (2.25)	0.002^*^	51.80 (3.65)	52.70 (2.40)	0.60
VVM2 construction 2 (T-scores)	41.20 (2.70)	44.70 (3.75)	0.11	48.50 (4.17)	48.50 (2.24)	1.00

Bonferroni corrected significant results are marked with asterisks (*).

Groups did not differ significantly during the pre-test (all *p* > 0.06).

### Mental Strategies

**Figure 4:**
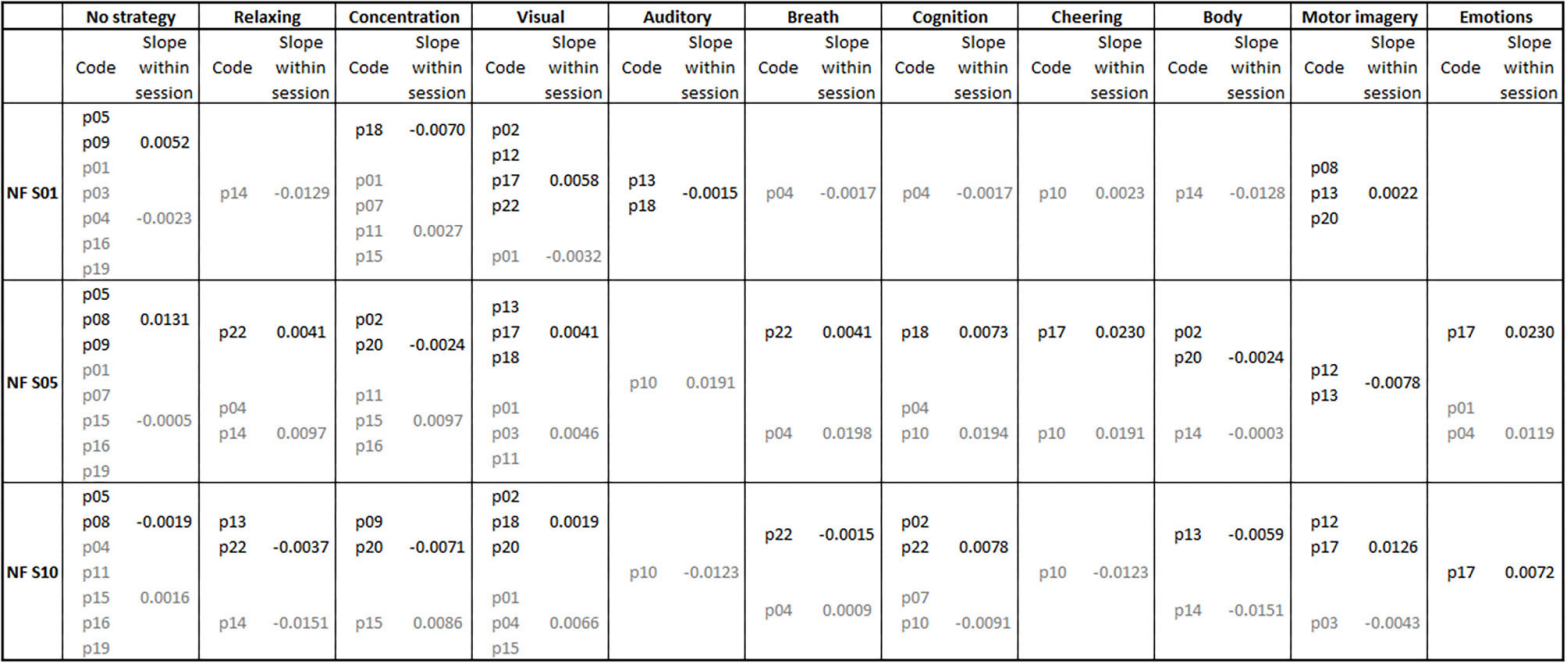
Mental strategies used during the first (NF S01), fifth (NF S05), and last (NF S10) neurofeedback training session, presented separately for each participant of the coherence up- (subject code in black font color) and down-regulation group (subject code in gray font color). Additionally, the average regression slopes of SMR coherence across feedback runs within neurofeedback training sessions are presented separately for the first, fifth, and tenth neurofeedback session and the up- (slope values in black font color) and down-regulation group (slope values in gray font color). A positive slope indicates a linear increase in SMR coherence across feedback runs within the corresponding neurofeedback training session.

In Figure 4, the frequencies of the mental strategies used to control the feedback bars during the first, fifth and last NF training session are shown, separately for both groups. Statistical analysis revealed no significant differences in the number of individually reported mental strategies between groups (all *p* > 0.06).

Figure 4 also illustrates the average regression slopes of SMR coherence across feedback runs within the NF training sessions, in which the mental strategies were assessed, per used mental strategy. These regression slopes should be interpreted with caution since for some mental strategies only the regression slope of a single participant was considered, if only one participant reported to use this specific mental strategy during a training session. However, descriptively the effectiveness of some used mental strategies varies between training sessions. For instance, “No strategy” was effectively used by the up-regulation group to linearly increase SMR coherence during the first and fifth NF training session. During the last NF session, this strategy had an opposite effect and led in this group to a linear decrease of SMR coherence within the training session. The coherence up-regulation group was instructed to use motor imagery strategies (Buch et al., 2012). This strategy led to a linear increase in SMR coherence during the first and last NF training session, but to a decrease during the fifth NF training session. Note that only a small number of participants of the up-regulation group really followed the instruction to imagine movements. Body movements led to a decrease in coherence in both groups. Using visual strategies, the up-regulation group was able to increase coherence within training sessions. The mental strategy “Concentration” led to an opposite effect in both groups (decrease in coherence in the up-regulation group and increase in coherence in the down-regulation group).

## Discussion

In the present study, we investigated the effects of NF training, in which the EEG connectivity in the SMR frequency range between central and posterior brain regions was used as feedback signal, on EEG power and coherence measures within and between 10 NF training sessions and cognitive performance. Additionally, we addressed the question whether participants are able to increase and decrease EEG coherence during NF training. Concerning the question whether NF users are able to up- and down-regulate EEG coherence voluntarily between two brain regions, we found different results when analyzing within- and between-sessions changes. Additionally, we analyzed mental strategies to control the feedback signal during NF training to reveal possible links between mental strategies and NF training success.

The NF training protocol, in which the SMR coherence should be up-regulated, led to significant increases in SMR coherence across the NF training sessions (between-session changes) as well as to a linearly increase in SMR coherence within the NF training sessions. This result indicates that a coherence up-regulation training led to stable between-session EEG changes (Klimesch, 1999), since SMR power also increased across the NF training sessions over Cz and CPz. A further indication for between-session changes in the EEG due to coherence-based NF training is the fact that we also found concomitant changes in SMR coherence assessed during the resting measurements with open eyes before and after 10 NF training sessions. Hence, a coherence up-regulation protocol led to stable changes in the background EEG as assessed during rest. Mottaz et al. (2018) also report that participants are able to up-regulate coherence. In that study, participants received feedback about functional connectivity in the motor cortex in the alpha frequency range (8–12 Hz). In contrast to the present finding, Mottaz et al. (2018) only found within session changes but no between session changes in brain connectivity during the NF training. However, they also report on linear between session increase in functional brain connectivity assessed during a resting measurement with open eyes performed before each of the 8 NF training sessions in participants who improved motor function (Mottaz et al., 2015, 2018), which is in line with the present findings. Kajal et al. (2017) showed that participants are able to up- and down-regulate inter-hemispheric connectivity of motor brain areas in the SMR frequency range during NF training. However, they only performed one session of NF training and, therefore, no predictions concerning possible between session changes are possible (Kajal et al., 2017). Studies reporting on QEEG-based coherence training do not report on the within- or between-session changes in EEG activity (Walker et al., 2002).

Many NF training studies, in which the amplitude of the SMR should be increased over one electrode position, observed within-session changes in SMR power but no between-session changes (Vernon et al., 2003; Vernon, 2005; Ros et al., 2010; Kober et al., 2015a,b, 2017b, 2019; Reichert et al., 2016). SMR-based NF studies that report on between-session changes either used ratios of the power within two or more frequency bands or relied on relative power changes limiting the evaluation of changes in absolute SMR power values between sessions (Gruzelier et al., 2010; Zambotti et al., 2012; Kober et al., 2013; Ros et al., 2013; Schabus et al., 2014, 2017). Generally, in the NF literature it is disputed whether NF training should lead to within- or between-session changes in the EEG (Enriquez-Geppert et al., 2017). When modulating for instance pathological EEG patterns, it is necessary to reach between-session or tonic changes in the EEG (Kropotov, 2009; Gruzelier, 2014b). When the aim of a NF training is to learn to voluntarily modulate a specific EEG signal at a given time, for instance when performing a cognitive task, it would make sense to be able to evoke task-related within-session changes (Dempster and Vernon, 2009; Kober et al., 2015b). The learned ability of participants to be able to modulate the EEG activity at a given time might not be necessarily related to between-session changes in the EEG. For instance, self-instruction to focus on a task (e.g., controlling the feedback bars during NF training) can be used for very specific purpose, when solving particularly important tasks (e.g., reaching a concentrated and focused mental state to improve cognitive performance), but is not necessarily a state one wants to be continuously in (e.g., trying to relax or to let your mind drift when not trying to solve specific tasks). The results of the present study show that in contrast to an SMR-based amplitude protocol, a coherence up-regulation protocol leads to between-session changes in SMR coherence and SMR power during NF training. When training to increase coherence between two brain regions, the coherence only goes up when the SMR rhythm changes in a comparable way over two different electrode positions (if there is a linear relationship of the two signals in the SMR frequency range), in our study, Cz and CPz. Hence, neurons that generate the signal recorded over Cz and neurons that generate the signal recorded over CPz were trained to fire synchronously probably leading to neuronal plasticity processes and consequently to between-session changes in the EEG, according to the Hebbian principle (Ros et al., 2014). Modulating the coherence between two brain regions activates neuronal networks producing the EEG signals at both electrode positions and, consequently, lead to more stable between-session changes in EEG activity due to neuronal plasticity processes (Elmer and Jäncke, 2014; Ros et al., 2014).

Up-regulating the coherence by means of NF training also led to changes in resting EEG activity. Kober et al. (2017b) also found changes in EEG resting measurements with open eyes after 10 sessions of amplitude-based SMR NF training. These findings also support the occurrence of between-session changes in the EEG due to NF training. Other NF studies that also provide feedback about the amplitude of a specific EEG frequency over one electrode position also demonstrated carry-over effects of NF training on baseline / resting measurements (Hanslmayr et al., 2005; Escolano et al., 2011; Zoefel et al., 2011; Ros et al., 2013; Gruzelier, 2014b). It seems as if both, amplitude and coherence NF training can affect the EEG as assessed during resting measurements.

Coherence down-regulation was not feasible for participants within training sessions nor between training sessions. Although there were no significant changes in SMR coherence within training sessions, SMR power decreased within training sessions over Cz and increased over CPz. This might indicate that participants were somehow able to modulate SMR power in the opposite direction at the two electrode positions. However, it might be generally more difficult to down-regulate coherence measures than to up-regulate them (Tinius and Tinius, 2000). To decrease EEG coherence, it is necessary to produce an unspecific and desynchronized EEG activity at the two recording sites (e.g., Cz and CPz). To reduce coherence, it is not important whether SMR power changes at Cz or at CPz, as long as SMR power does not change in the same way at both electrode positions at the same time. Additionally, it is a matter of future investigation to determine whether the coherence during a baseline or resting measurement before the start of the NF training might be a predictor of the coherence down-regulation success during NF training (Reichert et al., 2015; Yamashita et al., 2017). If a NF user already shows a low coherence at the beginning of the NF training, a further reduction of the coherence might be impossible. Other EEG studies in which participants successfully increased and decreased coherence values used QEEG in clinical samples, which showed pathological hyper- or hypo-connectivity (Walker et al., 2002; Coben, 2007; Thornton and Carmody, 2009; Coben et al., 2014, 2018). In our healthy sample of young adults, a linear and significant down-regulation of SMR coherence was not possible within or between NF training sessions.

As already mentioned, SMR coherence training also led to changes in SMR power. The coherence up-regulation group that successfully up-regulated coherence within and across training sessions also showed concomitant increases in SMR power recorded over Cz and CPz across training sessions but no linear changes in SMR power within training sessions. The coherence down-regulation group showed a significant linear decrease in SMR power within training sessions at Cz and a concomitant increase at CPz, but no significant changes in SMR power across training sessions. We did not only find significant changes in the target feedback frequency range (SMR), the control frequencies theta and beta also changed during coherence-based NF training. Theta power changed in an unspecific manner over Cz in both groups. No significant changes in theta power were observed over CPz. The up-regulation group showed a linear increase in beta power within training sessions over Cz but not over CPz, while the down-regulation group showed a linear increase in beta power within training sessions over both electrode positions. No significant changes in beta power across training sessions were observed in the up-regulation group. The down-regulation group showed an increase in beta power between sessions at Cz. To sum up, both groups showed some concomitant changes in the control frequencies. In prior amplitude-based SMR NF training studies, in which SMR amplitude could be voluntarily increased within training sessions, the control frequencies theta and beta did not change during NF training, which was interpreted as a sign of band specificity (Zoefel et al., 2011; Kober et al., 2017b). Independence between frequencies observed in amplitude-based NF training studies is not necessarily informative about changes in coherence between different electrode sites. It might be possible to increase the power at a specific electrode position and within a specific frequency-range (what one would interpret as a specific effect, Zoefel et al., 2011) while increasing or decreasing coherence between several other electrode sites and across several frequency bands. However, the observed changes in the control frequencies theta and beta do not seem to be systematically linked to changes in SMR coherence or SMR power within and between training sessions in the present study.

The coherence up-regulation group showed significant improvements in verbal short-term memory tasks (VVM2 construction 1, Digit Span forward) and working memory (Digit Span backwards) after the NF training compared to the pre-test. This is in line with prior single case reports of neurologic patients, in which positive effects of QEEG-based coherence NF training on memory function were reported (Thornton, 2000). In prior NF studies, in which the SMR amplitude, e.g., over Cz, should be increased, improvements in verbal memory functions were observed after SMR-based NF training, too (Vernon et al., 2003; Lévesque et al., 2006; Gruzelier, 2014a; Kober et al., 2015a,b). Improvements in cognitive functions due to SMR amplitude NF training were linked to a concomitant reduction in sensorimotor interferences, which might disturb cognitive processing (Sterman, 1996, 2000; Kober et al., 2015b; Reichert et al., 2016). This conclusion was based on findings of reduced coherence between motor areas (Cz) and more posterior areas (CPz) when performing a short-term memory task after NF training compared to a pre-measurement. Hence, these studies found a task-related reduction in coherence, which was linked to an improved cognitive processing (Kober et al., 2015b; Reichert et al., 2016). In contrast to the present findings, these SMR-based amplitude NF trainings found within-session changes in SMR power (Kober et al., 2015b; Reichert et al., 2016), while in the present study SMR power increased between NF training sessions and not task-related or within training sessions. Hence, our results indicate that between-session increases in EEG coherence and SMR power due to coherence-based NF training also lead to memory improvements. This does not necessarily contradict prior findings. Task-related decreases in SMR coherence when performing a memory task, which is linked to improved cognitive performance (Sterman, 1996, 2000; Kober et al., 2015b; Reichert et al., 2016), might be even easier when the overall SMR coherence is higher (after a between-session increase in SMR coherence). Additionally, in patients with memory deficits such as Alzheimer's disease or mild cognitive impairment, the EEG coherence in the tonic EEG (assessed during resting measurements) is pathologically decreased (Babiloni et al., 2016). Using EEG coherence training to up-regulate brain connectivity at rest might be a useful tool to improve memory functions in the future.

The coherence down-regulation group showed no significant changes in memory function after NF training compared to a pre-test. This group was also not able to modulate SMR coherence in the desired direction during NF training and did not show between-session changes in SMR coherence or power. Whether successful down-regulation of SMR coherence during NF training would have affected cognitive function remains open.

The analysis of the individually reported mental strategies used to control the feedback bars during NF training revealed no significant differences between groups, although both groups received different instructions on how to control the bars. The coherence up-regulation group was instructed to use motor imagery strategies (Buch et al., 2012). As can be seen in Figure 4, only about a fifth of this group really applied this strategy, with varying degrees of success. The down-regulation group was instructed to be mentally focused and physically relaxed at the same time (Kober et al., 2015b; Reichert et al., 2016). Four out of ten participants reported the strategy “Concentration,” which represents a focused state, during the first NF training session. However, over the course of the training participants of the down-regulation group used this strategy less often. During the 10^th^ NF training session, only one participant of this group reported to use the strategy “Concentration.” This is in contrast to prior amplitude-based NF training studies in which “Concentration” is one of the most frequently used mental strategies, although this strategy did not turn out to be the most effective one to increase SMR amplitude (Kober et al., 2013, 2017c; Autenrieth et al., 2020). The strategy “Relaxing” was only mentioned by 1-2 participants of this group per session. Hence, contrary to our expectation, the use of the typical instruction to increase SMR amplitude (to be mentally focused and physically relaxed at the same time), which lead to a concomitant decrease in SMR coherence in prior studies (Kober et al., 2015b; Reichert et al., 2016), did not lead to a successful down-regulation of SMR coherence in the present study.

We examined mental strategies three times after enough training might have shaped it, so that strategy changes driven by learning might be tracked. However, we did not find a clear connection between the used mental strategy and NF learning, as it was found in prior amplitude-based NF studies (Nan et al., 2012; Kober et al., 2013, 2017c; Autenrieth et al., 2020). When having a closer look at the regression slopes of SMR coherence within the NF training sessions, in which the mental strategies were assessed, the effectiveness of the majority of used mental strategies seems to vary between training sessions. Reporting to use no specific mental strategy during NF training turned out to be a successful strategy to increase SMR amplitude (Kober et al., 2013). For up-regulating SMR coherence, reporting “No strategy” was effective during the first and fifth NF training session but not during the last NF training session. Using visual strategies seemed to be the only mental strategy that led to an increase in SMR coherence in the up-regulation group in the first, fifth and last NF session. However, the results of the analysis of mental strategies should be interpreted with caution since one participant could report more than one mental strategy per session and it is not clear for how long a specific mental strategy was used during a training session, when participants switched strategies during training.

A reason for the missing link between the mental strategies used and NF success might be that NF learning is assumed to correspond to associative learning (Strehl, 2014). Generally, associative learning is impervious to consciousness while the use of what one may call strategy is a conscious and voluntary process. Another reason might be that the mental strategies connected to coherence training are completely different and produce other introspection than up-regulation of SMR amplitude or power. For the present study, it remains open whether the down-regulation group was not successful in reducing SMR coherence during NF training because they did not use proper mental strategies or because of other reasons. Overall, our results are in line with prior amplitude-based NF studies showing that participants are using a variety of different mental strategies during NF training (Nan et al., 2012; Kober et al., 2013, 2017c; Davelaar et al., 2018; Autenrieth et al., 2020). Based on the present explorative analysis of the used mental strategies, one cannot conclude which strategy works best to up- or down-regulate SMR coherence.

### Limitations

One limitation of the present study is the small sample size. With *N* = 10 per group, we can only reveal large effects. In future studies, the training duration may be increased as well. Ten NF training sessions might not be enough to uncover reliable effects and transfer of acquired NF functions (Kropotov, 2009; Strehl, 2014; Enriquez-Geppert et al., 2017). Additionally, results of our training protocol, in which EEG coherence between Cz and CPz was used as feedback signal, may not be generalizable to other coherence-based NF protocols, in which, for instance, the inter-hemispheric coherence is trained (e.g., Kajal et al., 2017). Another point is that the up- and down-regulation group received different instructions on how to modulate their brain activity in the desired direction. The up-regulation group was instructed to increase the bar in the middle of the screen by using for instance motor imagery. The down-regulation group was instructed to decrease the bar in the middle of the screen by being mentally focused and physically relaxed at the same time. These instructions were chosen based on prior studies (Buch et al., 2012; Kober et al., 2015b; Reichert et al., 2016). Although the groups received different instructions, the analysis of the individual reports of participants about the used mental strategies during NF training revealed that there was no difference in the used mental strategies between groups. Hence, differences in changes in SMR coherence and SMR power within and between NF training sessions between groups cannot be merely due to differences in instructions given to participants before the NF training. Future studies should also include further control groups. For instance, including a sham control group receiving fake feedback might help to better understand the specificity of the effects found in the present study as well as to reveal possible placebo effects (Thibault et al., 2017; Ros et al., 2019). Our results in healthy young adults might not be generalizable to older individuals or patient populations. Hence, future studies are necessary to investigate the effects of coherence-based NF training in elderly and in patient populations, for instance, in dementia patients, who generally show an altered EEG connectivity compared to healthy older individuals (Babiloni et al., 2016).

### Conclusion

Here we show that up-regulation of SMR coherence during NF training was possible and led to within- and between-session changes in the EEG as well as to improvements in memory functions. Decreasing SMR coherence by means of NF training was not possible. Our results indicate that NF training might be used to increase brain connectivity in patients with pathological hypo-connectivity and associated memory problems in the future (Babiloni et al., 2016).

## Data Availability Statement

Ethical restrictions prohibit the authors from making the data set publicly available. Data are available from the corresponding author (SEK) after contacting the Ethics Committee of the University of Graz ( ethikkommission@uni-graz.at) for researchers who meet the criteria for access to confidential data. When the Ethics Committee of the University of Graz agrees, the readers can contact the corresponding author (Silvia Erika Kober) (silvia.kober@uni-graz.at) to request the data. With an approval of the Ethics Committee of the University of Graz, we confirm that data will be available upon request to all interested researchers.

## Ethics Statement

The studies involving human participants were reviewed and approved by the Ethics Committee of the University of Graz, Austria. The participants provided their written informed consent to participate in this study.

## Author Contributions

SEK and GW performed research, wrote the original draft, funding acquisition, and involved in data analysis and interpretation. SEK, CN, and GW contributed resources, software, and/or analytic tools. All authors were involved in designing the research and conceptualization. All authors contributed to the article and approved the submitted version.

## Conflict of Interest

The authors declare that the research was conducted in the absence of any commercial or financial relationships that could be construed as a potential conflict of interest.
