# Supplementary material for: Differential Effects of Up- and Down-Regulation of SMR Coherence on EEG Activity and Memory Performance: A Neurofeedback Training Study
Source: Front Hum Neurosci. 2020 Dec 23;14:606684. doi: 10.3389/fnhum.2020.606684 (PMC7793696; doi:10.3389/fnhum.2020.606684)
Supplement: Supplementary file 1 [file Table_1.DOCX]

Supplementary Material

**Article title:** Differential effects of up- and down-regulation of SMR coherence on EEG activity and memory performance: A neurofeedback training study

**Silvia Erika Kober^1,2^, Christa Neuper^1,2^, Guilherme Wood^1,2^**

^1^ Institute of Psychology, University of Graz, Austria.

^2^ BioTechMed-Graz, Austria

# Supplementary Data – Magnitude squared coherence

In addition to the imaginary part of coherence, we also analyzed the magnitude-squared coherence, since participants received feedback on this coherence measure during real-time feedback. Since the magnitude squared coherence can be affected by volume conduction, we decided to report the results of the imaginary part of coherence in the main manuscript.

After EEG pre-processing, each neurofeedback (NF) run was cut in artifact free 1-sec epochs. FFT transformation was performed per epoch (Hanning window, 10%). Then the magnitude squared coherence was calculated for the channel pair Cz-CPz and average coherence values in the frequency range of 12-15 Hz were extracted per run.

For statistical analysis, we employed mixed-effects models with the linear fixed effects session (NF training session 1-10) and run (baseline run and 6 feedback runs) for the dependent variable magnitude squared SMR coherence between Cz and CPz separately for the SMR coherence up- and down-regulation group (Type I Analysis of Variance with Satterthwaite's method). Subjects, individual regression slopes across sessions and runs were included in the model as crossed random effects.

In Supplementary Table 1, the results of the mixed effect models for the dependent variable magnitude squared SMR coherence are summarized for both groups.

The coherence up-regulation group showed a linear increase in SMR coherence between sessions (Supplementary Figure 1b) as indicated by a significant main effect session (Supplementary Table 1). This might be a sign of between-session changes in EEG coherence in this group and is in line with the results of the imaginary part of coherence. The coherence down-regulation group showed no significant changes in SMR coherence, neither within nor between NF training sessions (Supplementary Figure 1), which is also in line with the results of the imaginary part of coherence, reported in the main manuscript. Supplementary Figure 2 shows changes in SMR coherence across feedback runs (within-session changes) separately for each of the 10 NF training sessions.

**Supplementary Table 1.** Results of the mixed-effects model with the linear fixed effects session (NF training session 1-10) and run (baseline run and 6 feedback runs) and the crossed random effects subjects, individual regression slopes across sessions and runs for the dependent variable magnitude squared SMR coherence during neurofeedback training, presented separately for the coherence up-regulation and down-regulation group

|  |  | Up-regulation group | | | Down-regulation group | | |
| --- | --- | --- | --- | --- | --- | --- | --- |
|  |  | *F (df, dfError)* | *MSE* | *p-value* | *F (df, dfError)* | *MSE* | *p-value* |
| Magnitude squared SMR coherence | Session | 8.597 (1,87.04) | 0.022 | 0.004** | 3.124 (1,87.98) | 0.008 | 0.081 |
|  | Run | 0.618 (1,53.95) | 0.002 | 0.435 | 1.290 (1,59.61) | 0.003 | 0.261 |
|  | Session*Run | 0.002 (1,527.62) | <0.001 | 0.966 | 0.069 (1,533.70) | <0.001 | 0.794 |

*Significant results are marked with * (*p< 0,05; **p<0,01; ***p<0,001)*

**
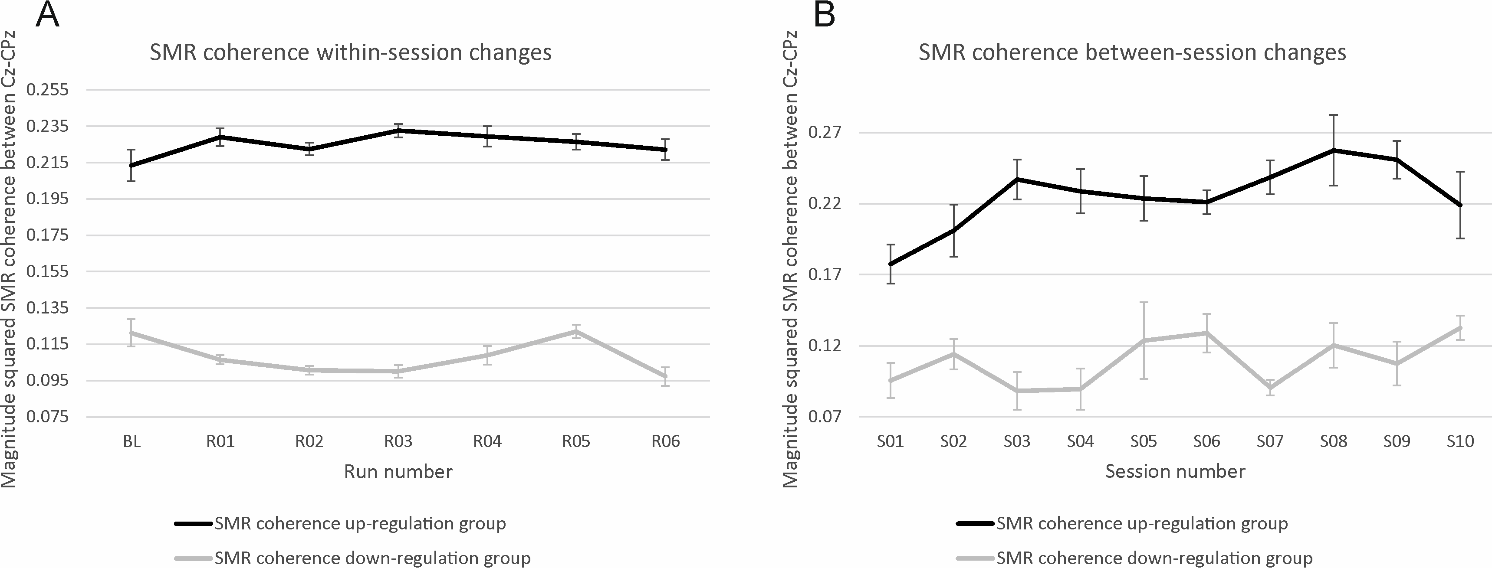
**

**Supplementary Figure 1.** Neurofeedback training performance. Changes in magnitude squared SMR coherence between Cz and CPz (a) within neurofeedback training sessions (averaged across all 10 training sessions) and (b) between neurofeedback training sessions (averaged across baseline run and six feedback runs per session), presented separately for the coherence up- and down-regulation group. Error bars represent the Cousineau-Morey transformed standard errors.

**
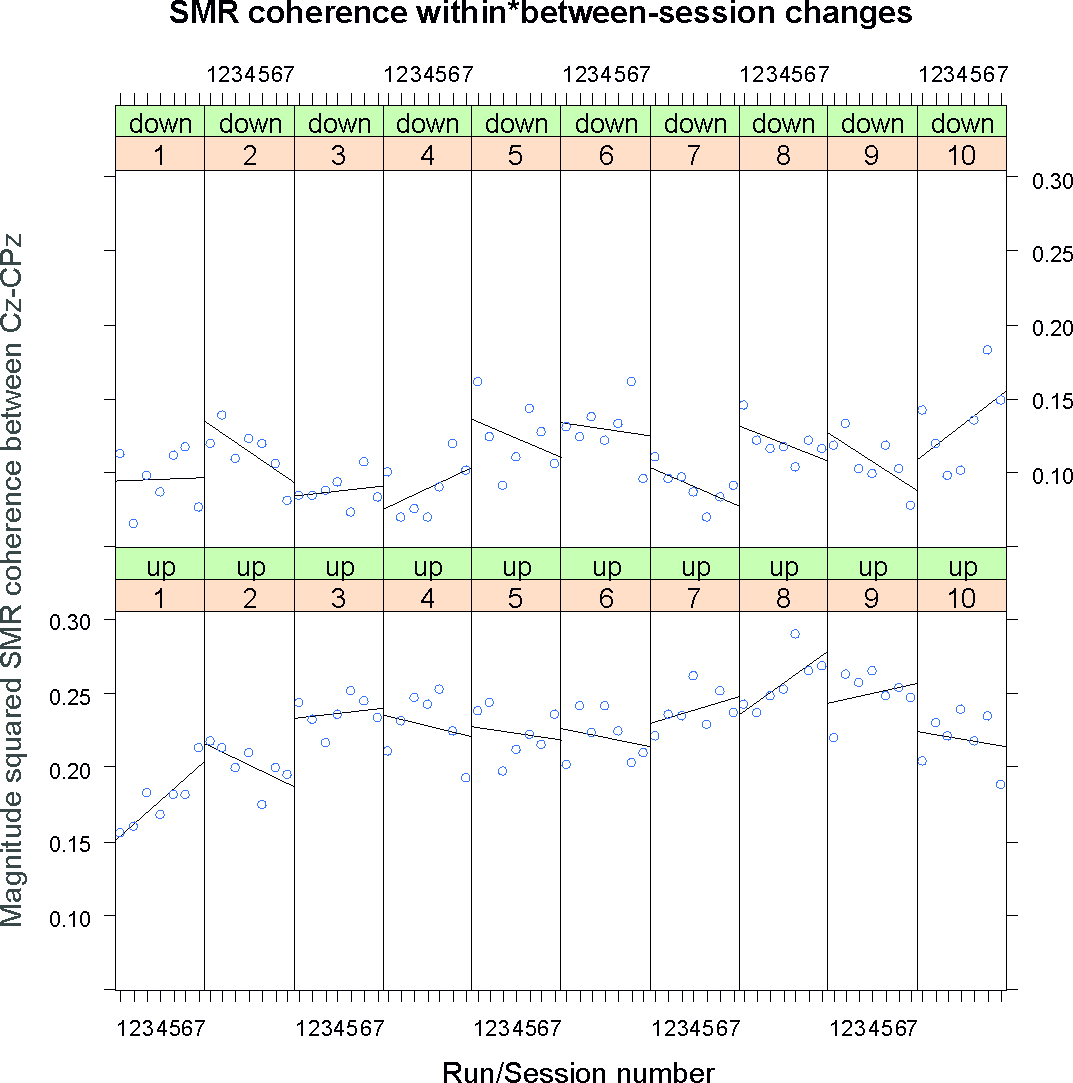
**

**Supplementary Figure 2.** Neurofeedback training performance per training session. Changes in magnitude squared SMR coherence between Cz and CPz across feedback runs (within-session changes, run 1 = baseline run, run 2 = 1st feedback run, run 7 = 6th feedback run), presented separately per neurofeedback training session (1-10) and group (down-regulation group: upper panel, up-regulation group: lower panel).
